# Yield and Quality Characteristics of *Brassica* Microgreens as Affected by the NH_4_:NO_3_ Molar Ratio and Strength of the Nutrient Solution

**DOI:** 10.3390/foods9050677

**Published:** 2020-05-25

**Authors:** Onofrio Davide Palmitessa, Massimiliano Renna, Pasquale Crupi, Angelo Lovece, Filomena Corbo, Pietro Santamaria

**Affiliations:** 1 Department of Agricultural and Environmental Science, University of Bari Aldo Moro, via Amendola 165/A, 70126 Bari, Italy; onofrio.palmitessa@uniba.it (O.D.P.); pietro.santamaria@uniba.it (P.S.); 2 Institute of Sciences of Food Production, National Research Council of Italy, via Amendola 122/O, 70126 Bari, Italy; 3 Council for Agricultural Research and Economics–Research Centre for Viticulture and Enology, Via Casamassima, 148, 70010 Turi, Italy; pasquale.crupi@crea.gov.it; 4 Department of Pharmacy-Drug Sciences, University of Bari Aldo Moro, Via Orabona, 4, 70125 Bari, Italy; angelo.lovece@uniba.it (A.L.); filomena.corbo@uniba.it (F.C.)

**Keywords:** broccoli, broccoli raab, cauliflower, hydroponic, mineral elements, nitrate, vitamins

## Abstract

Microgreens are gaining more and more interest, but little information is available on the effects of the chemical composition of the nutrient solution on the microgreen yield. In this study, three *Brassica* genotypes (*B. oleracea* var. *italica*, *B. oleracea* var. *botrytis*, and *Brassica rapa* L. subsp. *sylvestris* L. Janch. var. *esculenta* Hort) were fertigated with three modified strength Hoagland nutrient solutions (1/2, 1/4, and 1/8 strength) or with three modified half-strength Hoagland nutrient solutions with three different NH_4_:NO_3_ molar ratios (5:95, 15:85, and 25:75). Microgreen yields and content of inorganic ions, dietary fiber, proteins, α-tocopherol, and β-carotene were evaluated. Micro cauliflower showed the highest yield, as well as a higher content of mineral elements and α-tocopherol (10.4 mg 100 g^−1^ fresh weight (FW)) than other genotypes. The use of nutrient solution at half strength gave both a high yield (0.23 g cm^−2^) and a desirable seedling height. By changing the NH_4_:NO_3_ molar ratio in the nutrient solution, no differences were found on yield and growing parameters, although the highest β-carotene content (6.3 mg 100 g^−1^ FW) was found by using a NH_4_:NO_3_ molar ratio of 25:75. The lowest nitrate content (on average 6.8 g 100 g^−1^ dry weight) was found in micro broccoli and micro broccoli raab by using a nutrient solution with NH_4_:NO_3_ molar ratios of 25:75 and 5:95, respectively. Micro cauliflower fertigated with a NH_4_:NO_3_ molar ratio of 25:75 showed the highest dry matter (9.8 g 100 g^−1^ FW) and protein content (4.2 g 100 g^−1^ FW).

## 1. Introduction

Microgreens can be described as young and tender edible seedlings, produced by using seeds of different vegetable species, herbaceous plants, aromatic herbs, and wild edible plants, which are considered as ‘functional foods’ or ‘super foods’ because of their high nutritional value [1,2,3]. In recent years, microgreens have been increasingly used as basic ingredients in culinary preparations to obtain both sweet and savoury dishes with peculiar organoleptic traits [4]. Many species and local varieties of several botanical families, such as Brassicaceae, can be used for microgreen production [5,6]. The Brassicaceae family offers some of the most consumed vegetables worldwide and their seedlings have a generally good taste and high nutritional value. Many studies have been carried out on the nutritional propriety of different Brassicacea genotypes consumed as microgreens. For example, in a study by Xiao et al. [7], 30 genotypes of *Brassica* were analyzed in regards to the content of elements, while Sun et al. [5] analyzed the polyphenols profile of five Brassicacea species. Other authors [8] also evaluated the bioaccessibility of mineral elements and antioxidant compounds in some Brassicaceae microgreens.

Microgreens can be also used, instead of common vegetables, to reduce the daily intake of some elements when their restriction is required for health reasons. For example, Renna et al. [9] showed that a useful reduction in potassium can occur with three genotypes of microgreens in order to propose low-potassium vegetables for subjects affected by renal failure. Recently, many studies were carried out on microgreens in regards to the effect of artificial light on carotenoid content [10,11], growth and nutritional quality [12], antioxidant properties [13], and content of bioactive compounds [14]. Nevertheless, only a few studies have been done on the effects of nutrient solution strength on the growth and nutritional quality of microgreens [5]. On the other hand, the strength and optimal electric conductibility (EC) of the nutrient solution to maximize yield and content of bioactive compounds, and reduce fertilizer waste during microgreens production, are currently not clear. Some authors [15,16] used a nutrient solution with an EC of 1.12 mS cm^−1^, Kyriacu et al. [17] reported an EC of 0.3 mS cm^−1^ (but with organic substrate with an EC of 0.2 mS cm^−1^), Di Gioia et al. [18] indicated an EC of 1.3 mS cm^−1^, while an EC of 1.8 mS cm^−1^ was reported by Renna et al. [9]. In regards to the chemical composition, some authors [19,20] used a modified Hoagland nutrient solution containing 31.5, 24.2, 6.2, 30.0, 4.1, and 8 mg L^−1^ of N, K, P, Ca, Mg, and S, respectively. Di Gioia et al. [21] fertigated microgreens with nutrient solution containing 105.1, 117.4, 15.5, 92.5, 26.0, and 34.6 mg L^−1^ of N, K, P, Ca, Mg, and S, respectively, while Wieth et al. [22] used three concentrations (0, 50 and 100%) of a nutrient solution containing 214.2, 250.6, 43.7, 136.0, 26.5, and 35.0 mg L^−1^ of N, K, P, Ca, Mg, and S, respectively. The optimal nutrient solution is not clear and much work needs to be done in this area.

An important aspect of the nutritional quality of vegetable products is their nitrate (NO_3_) content. Nitrate per se is relatively non-toxic, but its reaction products and metabolites, such as nitrite, nitric oxide and N-nitroso compounds have raised concerns because of their implications for adverse health effects, such as methemoglobinemia or ‘blue baby syndrome’ [23]. In this context, it is interesting to highlight that hydroponic cultivation systems allow a reduction in nitrate content in leafy vegetables, without negatively affecting yield and quality, due to strategies such as partially replacing nitrate-based fertilizers with ammonium-based ones [24,25].

Few studies have been carried out until now on the influence of the NH_4_:NO_3_ molar ratio in nutrient solutions on mineral and phytochemical content of microgreens. Some authors [17,19,20] reported a NH_4_:NO_3_ molar ratio of 11:89 in nutrient solutions, while Wieth et al. [22] used a nutrient solution with a NH_4_:NO_3_ molar ratio of 9:91. At the same time, only the NO_3_ form was used in the nutrient solution by other authors [15,16,21]. Nevertheless, based on the studies carried out on mature vegetables [26,27,28], it is possible to hypothesize a potential reduction in nitrate content, as well as an improvement in nutraceutical value, in microgreens grown in varying NH_4_:NO_3_ molar ratios of the nutrient solution.

Starting from these remarks, the aims of the present study on three *Brassica* microgreens were to evaluate: (i) the effects of the nutrient solution strength on yield and quality parameters; and (ii) the physiological behaviour and some quality traits of microgreens fertigated with three different NH_4_:NO_3_ molar ratios.

## 2. Materials and Methods

### 2.1. Experimental Set-Up

Two experiments were conducted using a hydroponic system during the spring of 2015 in the greenhouse at the Experimental Farm ‘La Noria’ of the Institute of Sciences of Food Production of the Italian National Research Council (CNR), located in Mola di Bari (BA, Southern Italy). The first experiment was carried out from 16 March to 3 April, while the second one was carried out from 22 April to 5 May.

**Figure 1:**
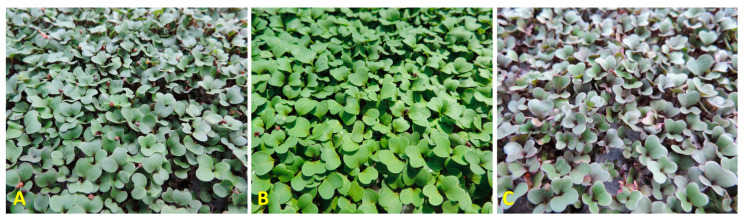
Genotypes used for producing microgreens: (**A**) broccoli, cultivar ‘Broccolo natalino’; (**B**) broccoli raab, local variety ‘Cima di rapa novantina’; (**C**) cauliflower, cultivar ‘Cavolfiore violetto’.

Three different genotypes of Brassicaceae were grown for both experiments: *Brassica rapa* L. subsp. *sylvestris* L. Janch. var. *esculenta* Hort, local variety ‘Cima di rapa novantina’ (broccoli raab); *Brassica oleracea* L. var. *italica*, cultivar ‘Broccolo natalino’ (broccoli); *Brassica oleracea* L. var. *botrytis*, cultivar ‘Cavolfiore violetto’ (cauliflower) (Figure 1). The seeds were purchased from ‘Riccardo Larosa Sementi’ (Andria, Italy) and their germination, tested at a constant temperature of 20 °C, was higher than 95%.

Microgreens were grown by using a hydroponic system with polyethylene terephthalate fiber pads (40 cm × 24 cm × 0.89 cm; Sure to Grow^®^; Sure to Grow, Beachwood, OH, USA) as a growing medium, which was placed on an aluminium bench (180 × 80 cm) with a slope of 0.05%. The seeds were uniformly broadcasted on the surface of the growing media using a seeding density of 4 seeds cm^−2^. The sown fiber pads were irrigated manually using a water-nozzle and were covered with a black polyethylene film until the germination was complete.

During the first experiment, three nutrient solutions (NSs), type-like Hoagland and Arnon [29], with different strengths (1/2 strength, 1/4 strength and 1/8 strength), prepared with rain water were used (Table 1). From germination until harvest, the NS was supplied for one minute in the morning and one minute in the afternoon.

For the second experiment, three half-strength NS with different ratios of NH_4_:NO_3_ were used (Table 2).

To prepare the nutrient solutions, fertilizers for hydroponic production were used. More specifically, the following salts were used: calcium nitrate, potassium nitrate, ammonium nitrate, potassium sulphate, magnesium sulphate, calcium chloride, and potassium dihydrogen phosphate. In order to obtain the element composition reported in Table 1 and Table 2, the amount of each salt was calculated, while also considering their titre and purity.

During the second experiment, being in late spring, the temperature in the greenhouse was higher than in the first experiment, for this reason another minute of fertigation was supplied at noon. During both the first and second experiment, after epicotyl emission, NSs were distributed by a drip tape line with pressure-compensated drippers (each with a delivery rate of 0.133 L min^−1^). An open cycle management was used; therefore, the drainage was collected but not reused. The experimental scheme used was split-plot where each plot was represented by the bench and each sub-plot was represented by a genotype.

### 2.2. Harvesting and Physical Analysis

Harvesting was carried out by cutting microgreens just above the growing media surface, when the first true leaves were at least 1 cm long. Within each experiment, three samples were considered for each experimental unit (genotype and treatment), and analysed as independent replicates. Each field replicate was obtained by harvesting three sub-samples within the same growing pad.

For both experiments and for each cultivar, we recorded how many days passed from sowing until: breaking seed integuments, radicle spillage, hypocotyl emission, cotyledons formation, first true leaf formation, second true leaf formation (true leaf was formed when it was at least 0.5 cm long). Immediately before the harvesting, other parameters were collected: presence of true leaves, leaf length (true leaf eventually present), shoot height and substrate coverage. To determine presence of true leaves, shoot height and leaf length, three random microgreens were selected for each sub-parcel. The substrate coverage included the distribution and microgreens overlap in the substrate. We used three different categories: 1—low; 2—good; 3—excessive. Each sub-parcel was observed at 30 cm, orthogonally from the growth plan and when possible, between the shoots to watch spare space, where we used category 1. If it was not possible to watch the growth media and there was not any overlap between the shoots, we used category 2, and category 3 was used when there was overlap between the shoots.

The harvested microgreens were weighed to determine the shoot fresh weight (FW) per unit area. The dry matter (DM) was measured in triplicate by oven-drying at 65 °C until a constant weight of the samples. The oven-dried samples were used for cation and anion content determination, while freeze-dried (ScanVac CoolSafe 55-9 Pro; LaboGene ApS, Lynge, Denmark) samples were used for chemical analysis.

### 2.3. Inorganic Ion Content

The content of inorganic ion was determined by ion exchange chromatography (Dionex DX120; Dionex Corporation, Sunnyvale, CA, USA) with a conductivity detector, as reported by D’Imperio et al. [17]. The content of Na^+^, K^+^, Mg^2+,^ and Ca^2+^ was determined in 1 g of dried sample, using an IonPac CG12A guard column and an IonPac CS12A analytical column (Dionex Corporation); the elution was performed with 18 mM of methanesulfonic acid (Thermo Scientific™ Dionex™, Waltham, MA, USA). Peaks identification and calibration were performed using the Multi Element IC Standard solution Fluka TraceCERT^®^, Supelco^®^ (Merck KGaA, Darmstadt, Germany). The contents of Cl^−^ and NO_3_^−^ were determined in 0.5 g of dried sample using an IonPac AG14 precolumn and an IonPac AS14 separation column (Dionex Corporation). The eluent consisted of 3.5 mmol·L^−1^ of sodium-carbonate (Thermo Scientific™ Dionex™, USA) and 1.0 mmol·L^−1^ of sodium-bicarbonate solution (Thermo Scientific™ Dionex™, USA), and 50 mL of the same eluent was used to extract the anions. Inorganic cation content determination was carried out in triplicate. Peaks identification and calibration were performed using the Multi Element IC Standard sol. IC-MAN-18 (6E) of Chem-Lab (Palin Corporation, Elderslie, UK).

### 2.4. Dietary Fiber Content

Dietary fiber content was determined according to AOAC methods [30] with a slight modification. First, a sample of lyophilized microgreen powder (250 mg) was boiled in 32.5 mL of H_2_SO_4_ 0.64 N for 10 min, adding a few drops of n-octanol as antifoam agent. The resulting insoluble residue was filtered, washed with warm distilled water, and boiled in 32.5 mL of KOH 0.56 N for 10 min. After filtering and washing the sample three times with acetone RPE, it was dried at 105 ± 2 °C for 1 h. Weight loss, corresponding to the raw fiber, was determined after cooling the sample at RT in a dryer. Then, ash content was determined by weighing the obtained residue before and after a strong heat treatment (550 °C for 3 h). Finally, fiber content was expressed relative to the fresh weight (FW). Crude protein was assessed by the micro-Kjeldahl method, with a nitrogen to protein conversion factor of 6.25, according to the AOAC method 976.05 [30]. Dietary fiber content determination was carried out in triplicate. All chemicals used were supplied by Sigma-Aldrich (Milan, Italy) and were of analytical grade.

### 2.5. Content of α-Tocopherol and β-Carotene

For α-tocopherol and pro-vitamin A expressed as β-carotene, the extraction procedure simultaneously extracts water-soluble vitamin (WSV) and fat-soluble vitamin (FSV). During the extraction process, samples were always protected from direct exposition to light and kept on ice to minimize vitamin degradation. Briefly, 0.050 g of each sample was first extracted with 7.5 mL of 1% BHA in ethanol and 500 μL of internal standard (86.82 μM trans-β-apo-8 carotenal) were added. Samples were placed in an ultrasound bath for 15 s and 180 μL of 80% KOH were added and heated for 45 min at 70 °C. Three milliters of water and 3 mL of hexane/toluene were added (10:8 v/v), and centrifuged at 1000× *g* for 5 min. The supernatant was recovered and the bottom solution was extracted with hexane/toluene at least two times. The phases were reunited and the solvent was evaporated under the nitrogen stream. It was recovered with 500 μL of acetonitrile/ethanol 1:1 for HPLC analysis. Separation and identification of lipophilic vitamins in microgreen extracts were carried out with a HPLC 1100 equipped with quaternary pump solvent delivery, thermostatic column compartment, and diode array detector (DAD) (Agilent Technologies, Palo Alto, CA, USA). The samples (20 μL) were injected onto a reversed stationary phase ZORBAX EC18 (Agilent Technologies) (5 μm (150 × 4.6 mm i.d.)), following an isocratic program with ethanol/acetonitrile 1:1 as mobile phase according to the method previously published by Xiao et al. [7]. Stop time was set at 30 min with a re-equilibration time of 10 min corresponding to ~20 column volume (Vc = 0.52 mL). The column temperature was not controlled, while the flow was maintained at 1.2 mL/min. Diode array detection was between 250 nm and 650 nm and absorbance was recorded at 450 nm for β-carotene and 290 nm for α-tocopherol. Compounds identification was achieved by combining different information: positions of absorption maxima (λmax), the degree of vibration fine structure (% III/II), and retention times were compared with those from pure standards. To evaluate linearity, calibration curves with five concentration points for each compound were prepared separately. Calibration was performed by linear regression of peak-area ratios of the vitamins to the internal standard (β-apo-8′-carotenal) versus the respective standard concentration, obtaining R^2^ values of 0.9992 and 0.9999 for β-carotene and α-tocopherol, respectively. Finally, vitamins were quantified as mg of β-carotene and α-tocopherol per 100 g of microgreens. The determination of α-tocopherol and β-carotene content was carried out in triplicate. All chemicals used were supplied by Sigma-Aldrich (Milan, Italy) and were of analytical grade.

### 2.6. Statistical Analysis

The data were analysed by a two-way analysis of variance (ANOVA), using the general linear model procedure of SAS software (SAS Version 9.1, SAS Institute, Cary, NC, USA) and applying a split-plot design with genotype (G) and nutrient solution (NS) as main factors for all measurements. All means were compared using the Student–Newman–Keuls (SNK) test at *p* = 0.05, and standard deviation (SD) was also calculated. Significance of main factors and their interaction are reported in tables. Average values of main factors are reported in tables, while average values of significant interactions G x NS are showed by using histograms.

## 3. Results

### 3.1. First Experiment

**Figure 2:**
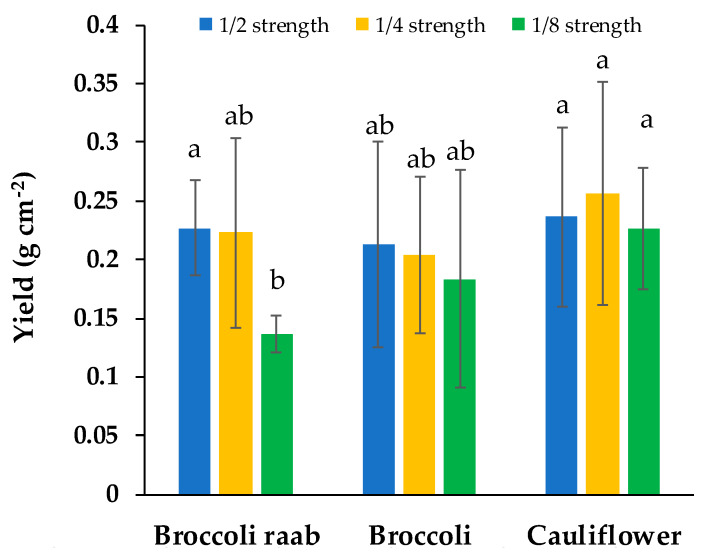
Yield of three genotypes of microgreens grown with three NS strengths: 1/2 strength, 1/4 strength and 1/8 strength (first experiment). Different letters indicate that mean values are significantly different, according to the SNK test (*p* = 0.05). Vertical bars represent ± standard deviation of mean values.

At harvest, broccoli raab showed twice the number of true leaves per seedling compared to other genotypes, while the average leaf length was about 1.28 cm, without any difference between genotypes, treatments and their interaction (Table 3). In regards to yield, broccoli raab fertigated with 1/8 strength NS showed an amount 43% lower compared with cauliflower, and 40% lower compared with broccoli raab fertigated with NS 1/2 (Figure 2). Microgreens fertigated with the 1/8 strength NS showed the lowest seedling height, which was 17% lower than those fertigated with 1/4 strength NS and 25% lower than those fertigated with 1/2 strength NS (Table 3). On the other hand, broccoli raab microgreen height was 9% lower compared with broccoli (Table 3).

The average values of development stage and density were, respectively, 3.0 and 4.0, without differences between genotypes, nutrient solution strength and their interaction (Table 4). Cauliflower showed a substrate coverage 27% lower than the other genotypes, while broccoli raab showed a value of substrate uniformity 43% higher than cauliflower (Table 4).

### 3.2. Second Experiment

Even in this experiment, broccoli raab showed twice the number of true leaves compared to broccoli and cauliflower, with leaves longer than 1 cm and seedling height 7% lower compared to the other species (Table 5). Cauliflower yield was 35% higher than broccoli raab and broccoli, beyond the chemical forms of nitrogen used (Table 5).

By using a NS with a NH_4_:NO_3_ 25:75 molar ratio, microgreens showed the highest content of Cl^−^ and K^+^. Cl^−^ was 75% higher in microgreens grown with a NH_4_:NO_3_ 25:75 molar ratio than other samples, while K^+^ was 6% and 19% higher in microgreens grown with a NH_4_:NO_3_ 25:75 molar ratio than NH_4_:NO_3_ 15:85 and 5:95 molar ratios, respectively. Between genotypes, broccoli showed a K^+^ content 11% higher than other genotypes (Table 6).

Microgreens grown by using a NS with a NH_4_:NO_3_ 15:85 molar ratio showed a SO_4_^2−^ content 13% higher than the other molar ratio (Table 6). Between the genotypes, cauliflower showed a SO_4_^2−^ content of 14% and 28% higher than broccoli and broccoli raab, respectively (Table 6). Ca^2+^ content was 14% higher in microgreens grown with the molar ratio NH_4_:NO_3_ 5:95 than 25:75, while the average Mg^2+^ content was 0.3 g 100 g^−1^ DW, without differences between genotypes, NH_4_:NO_3_ ratios and their interaction (Table 6).

**Figure 3:**
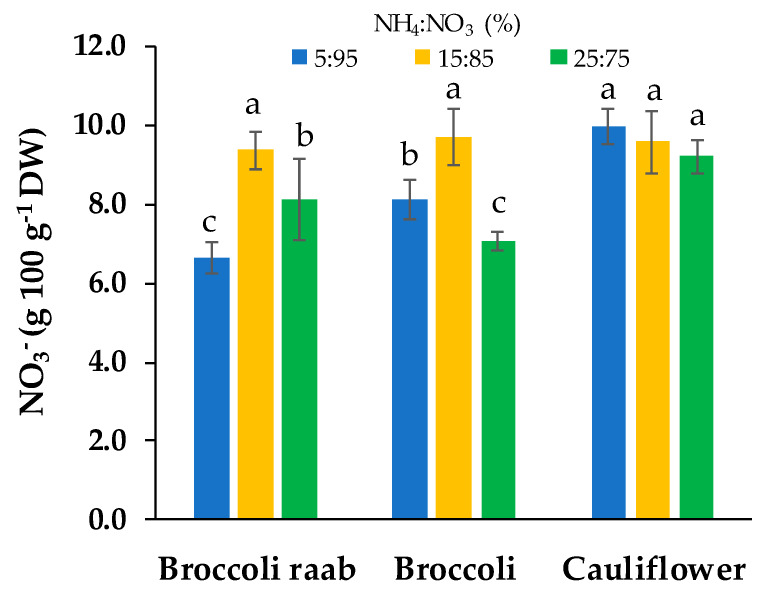
Nitrates (NO_3_^−^) content of three genotypes of microgreens grown by using a NS with three different NH_4_^+^:NO_3_^−^ (%) molar ratios: 5:95, 15:85 and 25:75. Different letters indicate that mean values are significantly different, according to the SNK test (*p* = 0.05). Vertical bars represent ± standard deviation of mean values.

Broccoli raab had the lowest and highest nitrate content with the molar ratio NH_4_:NO_3_ 5:95 and 15:85, respectively, while broccoli showed the lowest and highest nitrate content with NH_4_:NO_3_ 25:75 and 15:85, respectively. No differences were found in nitrate content in cauliflower by using different NH_4_:NO_3_ ratios (Figure 3).

**Figure 4:**
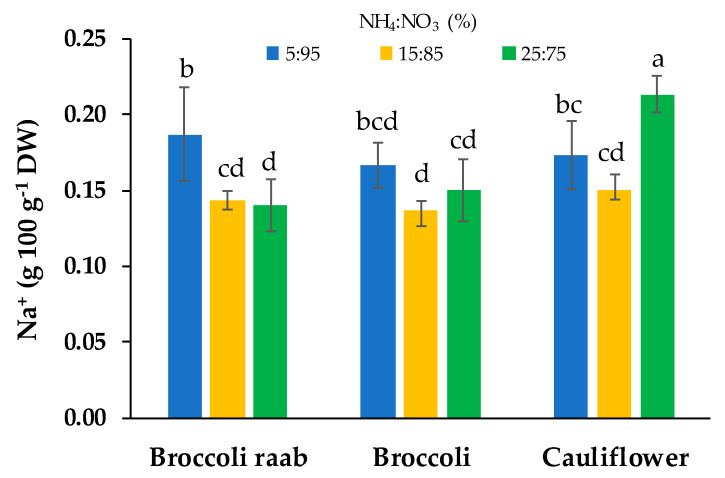
Sodium (Na^+^) content of three genotypes of microgreens grown by using a NS with three different NH_4_^+^:NO_3_^−^ (%) molar ratio: 5:95, 15:85 and 25:75. Different letters indicate that mean values are significantly different, according to the SNK test (*p* = 0.05). Vertical bars represent ± standard deviation of mean values.

Cauliflower grown using a NS with a NH_4_:NO_3_ 25:75 molar ratio showed the highest sodium content, which was 31% higher than the two other molar ratios used for the same genotype (Figure 4). Broccoli raab grown by using a NS with a NH_4_:NO_3_ 5:95 molar ratio showed a sodium content 36% higher than the other molar ratios of the same genotype. The average sodium content in broccoli was 0.15 g 100^−1^ DW, without differences between NH_4_:NO_3_ molar ratios (Figure 4).

**Figure 5:**
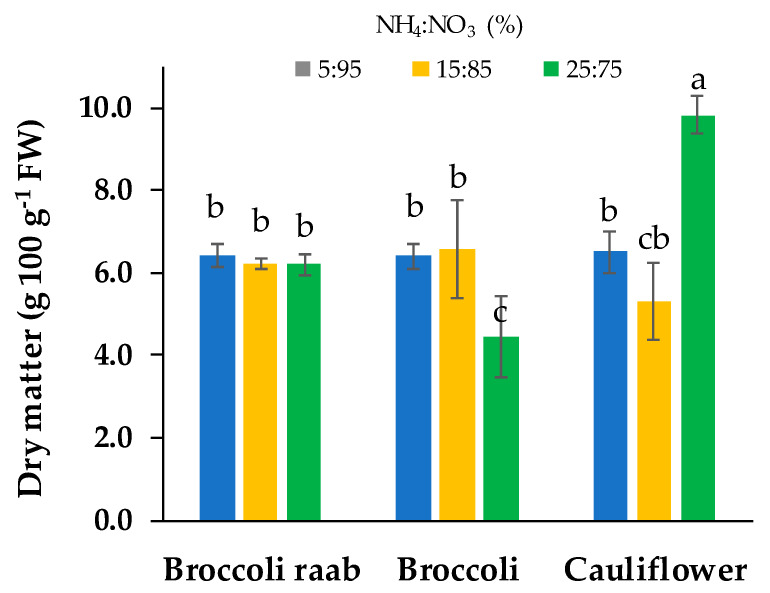
Dry matter content of three genotypes of microgreens grown by using a NS with three different NH_4_^+^:NO_3_^−^ (%) molar ratios: 5:95, 15:85 and 25:75. Different letters indicate that mean values are significantly different, according to the SNK test (*p* = 0.05). Vertical bars represent ± standard deviation of mean values.

The highest value of dry matter was obtained from cauliflower grown with a NH_4_:NO_3_ 25:75 molar ratio that resulted in 66% higher content than the two other molar ratios of the same genotype (Figure 4). Broccoli showed a dry matter content 31% lower with a NH_4_:NO_3_ 25:75 molar ratio compared to other molar ratios. The average content of dry matter in broccoli raab was 6.3 g 100^−1^ FW, without differences between NH_4_:NO_3_ molar ratios (Figure 5).

The average fiber content was 0.518 g 100 g^−1^ FW without significant differences between genotypes, NH_4_:NO_3_ molar ratios and their interaction (Table 7). Cauliflower showed an α-tocopherol content 194% higher than other genotypes, while broccoli raab showed a β-carotene content about 40% lower than other genotypes. The highest value of β-carotene was obtained with a NH_4_:NO_3_ 25:75 molar ratio that resulted in 40% higher content than the two other molar ratios (Table 7).

**Figure 6:**
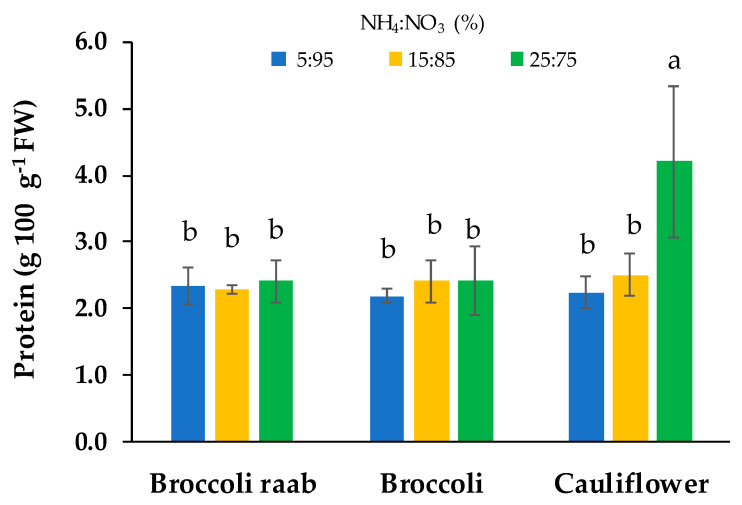
Protein content of three genotypes of microgreens grown by using a NS with three different NH_4_^+^:NO_3_^−^ (%) molar ratios: 5:95, 15:85 and 25:75. Different letters indicate that mean values are significantly different, according to the SNK test (*p* = 0.05). Vertical bars represent ± standard deviation of mean values.

As for protein content, cauliflower grown with a NH_4_:NO_3_ 25:75 molar ratio gave the highest value, which resulted in 79% higher content than the other nutrient solutions (Figure 6).

## 4. Discussion

In this study, we produced microgreens of some Brassicaceae genotypes by using a hydroponic system to evaluate the effects of element concentration and chemical form of nitrogen in the nutrient solution on yield and some quality traits. We conducted an exploratory experiment by using a NS type-like Hoagland and Arnon [29] but at three different strengths (1/2 strength, 1/4 strength and 1/8 strength). This, we started from the fact that some authors reported the use of a quarter-strength Hoagland nutrient solution [19,20], while other authors reported the use of a half-strength Hoagland nutrient solution [15,16] as well as three different strengths of nutrient solution [22]. Therefore, considering the short growth cycle of microgreens, we decided to also evaluate if nutrient concentration lower than half strength may satisfy seedling needs, without negatively affecting yield and other important parameters. In this context, it is important to highlight that the optimal choice of element concentration in the NS may allow one to reduce production costs and environmental impact. In the first experiment, we observed that growing parameters were not affected by NS strength (Table 4). In addition, yield was not affected by the NS strength except for broccoli raab, which showed a lower yield when 1/8 NS was used (Figure 2) and for this cultivar, the growth rate was faster than for broccoli and cauliflower (Table 4). On average, we found that seedling height significantly decreased when passing from NS at 1/2 strength to NS at 1/8 strength (Table 3). Considering that the harvesting of microgreens is usually done manually, the higher the seedling height, the easier the harvesting can be made. Therefore, for the second experiment, we decided to use a NS at 1/2 strength but with three different NH_4_:NO_3_ molar ratios to evaluate the effect of another aspect of fertigation on physiological behaviour and some quality traits of different Brassicaceae microgreens. The choice of NS at 1/2 strength instead of other ones was also made by considering the higher temperature and photosynthetic photon flux (PPF) forecasted for the second experiment than the first one. Effectively, the rate of nutrient uptake was related to current seedling nutrient demand, positively correlated with PPF and air temperature [31].

By changing the NH_4_:NO_3_ molar ratio, no differences were found on yield and growing parameters (Table 5), while significant differences were found in regards to dry matter and content of inorganic cations, proteins and β-carotene (Table 6 and Table 7). For dry matter, nitrates, sodium and proteins, we observed important interactions between genotypes and the molar ratio between the chemical forms of nitrogen. The most abundant cation in all the microgreens samples was K^+^, followed by Ca^2+^, Mg^2+^ and Na^+^, while, in regards to anion content, NO_3_^−^ was followed by SO_4_^2−^ and Cl^−^ (Table 6). A similar mineral composition was observed in previous studies [17,32]. In regards to the differences in nitrates content (Figure 3), Santamaria [23] reported that the large variation in nitrate accumulation among plant species could be associated with genetic factors. At the same time, different genotypes may show different nitrate uptake, translocation and accumulation in the vacuoles of mesophyll cells [33]. In agreement, we observed that by using a NS with the NH_4_:NO_3_ molar ratio of 5:95, broccoli raab showed a nitrate content lower than other NH_4_:NO_3_ molar ratios, while broccoli showed the lowest nitrates content when the NS with the NH_4_:NO_3_ molar ratio of 25:75 was used (Figure 3). At same time, no differences in nitrates content were found by changing the NH_4_:NO_3_ molar ratio in cauliflower (Figure 3). These results suggest that the nitrate content in different *Brassica* microgreens can be affected by the interaction between genotypes and the NH_4_:NO_3_ molar ratio in the NS. This is in agreement with Dikson and Fisher [34], who observed that genotypes had a central role in anion and cation uptake by varying root zone pH. In the same way, during this study, changing the NH_4_:NO_3_ molar ratio and substrate/root zone pH changes influenced cation and anion (nitrates) uptake differently for each genotype.

From a commercial point of view, it could be interesting to evaluate the nitrate content in microgreens observed in our study in relation to the tolerable levels of nitrates in foodstuffs. On average, we found a content of 5051, 4816 and 6249 mg NO_3_^−^ kg^−1^ FW, respectively for broccoli raab, broccoli and cauliflower (processed data from Table 6). It is important to note that for Brassicaceae species the European Regulation (EU) No 1258/2011 [35] reports maximum levels of nitrate only for the “rucola” group (*Eruca sativa*, *Diplotaxis* spp, *Brassica tenuifolia*, *Sisymbrium tenuifolium*). European Regulation fixed a maximum level of 7000 mg NO_3_ kg^−1^ FW for “rucola” vegetables harvested from 1st of October to 31st of March (the period of our study), and a maximum level of 6000 mg NO_3_ kg^−1^ FW in the other year period. Considering these maximum levels, our results suggest that by changing the NH_4_:NO_3_ molar ratio in the NS, it is possible to produce microgreens of broccoli raab, broccoli and cauliflower without negatively affecting an important commercial characteristic such as the nitrate content.

In regards to the nutritional quality, we found that all three genotypes of *Brassica* microgreens showed a high content of mineral elements (Table 6). This is agreement with several authors [17,32,36,37] confirming that microgreens can be considered as a good source of minerals in the human diet. Apart from the content of mineral elements, microgreens can provide higher amounts of other nutrients compared to their mature leaf counterparts [1]. To this end, we found that 100 g of mature cauliflower supplies about 2 g of fibers, 1.92 g of proteins and 0.08 mg of α-tocopherol [38]. The same serving size of mature broccoli supplies 2.6 g of fibers, 2.82 g of proteins and 0.78 mg of α-tocopherol [39], while 100 g mature broccoli raab supply 2.7 g of fibers, 3.17 g of proteins, and 1.62 mg of α-tocopherol [40]. Results of the present study show a fiber content (Table 7) much lower than mature plants independently of genotypes and the NH_4_:NO_3_ molar ratio. Therefore, according to Renna et al. [9], microgreens of this study can be considered as a low content fiber food for subjects with gastrointestinal disorders, such as bowel colon syndrome. Regarding protein content, microgreens showed values similar to mature *Brassica* vegetables with the exception of micro-cauliflower fertigated by using a NS with a NH_4_:NO_3_ molar ratio of 25:75, which showed a higher protein content than mature cauliflower. This, could be due to the fact that the NH_4_:NO_3_ molar ratio of 25:75 caused an increase in dry matter content compared with other treatments and proteins are one of the major constituents of the dry matter [41].

α-Tocopherol is the most common and biologically active form of vitamin E. Effectively, although the term vitamin E can refer to different types of tocopherols and tocotrienols, it should be considered the selective degradation and excretion of other vitamin E forms and the selective retention of α-tocopherol, mediated by the hepatic α-tocopherol transfer protein (α-TTP) [42]. In our study, we observed a higher α-tocopherol content, independently of the NH_4_:NO_3_ molar ratio, in microgreens than in the mature counterparts, especially in micro cauliflower (Table 7). α-Tocopherol represents part of the fat-soluble antioxidant system of the cell, since it terminates the chain reaction of lipid peroxidation. Vitamin E deficiency is associated with a progressive necrosis of the nervous system and muscle. In this context, it is important to note that the recommended dietary allowance (RDA) of vitamin E (α-tocopherol) for people aged 14 years and over, including pregnant women, is 15 mg per day [42]. Therefore, 100 g of microgreens produced in this study can satisfy about 70, 34 and 13% of the RDA, respectively, for micro cauliflower, micro broccoli and micro broccoli raab.

β-Carotene is the principal pro-vitamin A carotenoid considering that its symmetrical chemical structure always provides vitamin A regardless of the metabolic process. Other forms of provitamin A are α-carotene, γ-carotene and β-cryptoxanthin. β-Carotene is the most abundant dietary carotenoid present in yellow-orange fruits and vegetables, and green leafy vegetables. In humans, it plays a potent antioxidant role known to prevent oxidative damage to biological membranes by quenching free radicals [42]. Mature cauliflower lacks β-carotene [38], while 100 g of mature broccoli and broccoli raab contain 0.36 and 1.57 mg of β-carotene, respectively [39,40]. Therefore, results of the present study show a higher β-carotene content in microgreens than the mature counterparts, especially by using a NH_4_:NO_3_ molar ratio of 25:75 (Table 7). In a study aimed to evaluate the nutrient composition of ten culinary microgreens, Ghoora et al. [43] found a β-carotene content ranging from 3.1 to 9.1 mg 100 g^−1^ FW. Our results are in agreement with these authors, confirming that microgreens can be considered a good source of β-carotene, although the amount can vary depending on genotype.

## 5. Conclusions

All three *Brassica* genotypes can be considered suitable for microgreen production, although micro cauliflower showed the highest yield, as well as a higher content of some mineral elements and α-tocopherol compared to other genotypes, while micro broccoli raab showed the fastest growth rate. The use of a nutrient solution type-like Hoagland and Arnon at half strength allowed us to obtain both high yield and desirable seedling height. By changing the NH_4_:NO_3_ molar ratio in the nutrient solution, no differences were found on yield and growing parameters, while the highest β-carotene content was found by using a nutrient solution with a NH_4_:NO_3_ molar ratio of 25:75. The lowest nitrate content was found in micro broccoli by using a nutrient solution with a NH_4_:NO_3_ molar ratio of 25:75 and in micro broccoli raab by using a nutrient solution with a NH_4_:NO_3_ molar ratio of 5:95. Micro cauliflower grown by using a nutrient solution with a NH_4_:NO_3_ molar ratio of 25:75 showed the highest dry matter and protein content. From a commercial point of view, we highlight the possibility of producing microgreens of broccoli raab, broccoli and cauliflower by changing the NH_4_:NO_3_ molar ratio in the nutrient solution without negatively affecting an important characteristic such as the nitrate content. It could be interesting to assess the optimal strength and NH_4_:NO_3_ molar ratio of the nutrient solution to obtain the best yield performance and quality for microgreens of other botanic families. Moreover, quality evaluation during cold storage of fresh-cut microgreens obtained by using nutrient solutions with different strengths and NH_4_:NO_3_ molar ratios may be a possible next goal.

## Figures and Tables

**Table 1:** Characteristics of the nutrient solutions (NS) used during the first experiment.

	NS Strength		
	1/2	1/4	1/8
	(mg L^−1^)		
N-NO_3_	100	50	25
N-NH_4_	5	2.5	1.25
K	117	58.5	29.25
P	16	8	4
Mg	24	12	6
Ca	86	43	21.5
Cl	0	0	0
S	31	15.5	7.75
pH	6.5	6.5	6.5
EC (mS cm^−1^)	1.37	0.77	0.43

**Table 2:** Characteristics of the nutrient solutions used during the second experiment.

	Molar Ratio NH_4_:NO_3_ (%)		
	5:95	15:85	25:75
	(mg L^−1^)		
N-NO_3_	100	90	80
N-NH_4_	5	16	26
K	117	117	117
P	16	16	16
Mg	24	24	24
Ca	86	86	86
Cl	0	0	20
S	31	57	71
pH	6.3	5.9	5.8
EC (mS cm^−1^)	1.12	1.42	1.40

**Table 3:** Main effects of genotypes and nutrient solution strength on number and length of true leaves, yield and seedling height of microgreens (first experiment).

	True Leaves	Leaves Length	Yield	Seedling Height
	Number Seedling^−1^	cm	g cm^−2^	cm
**Genotype (G)**				
Broccoli raab	2.00 ± 0.30 a	1.43 ± 0.24	0.20 ± 0.02 b	8.60 ± 1.60 ab
Broccoli	1.00 ± 0.10 b	1.17 ± 0.32	0.20 ± 0.05 b	9.70 ± 1.10 ab
Cauliflower	1.00 ± 0.10 b	1.23 ± 0.19	0.24 ± 0.02 a	9.10 ± 1.40 b
**Nutrient solution strength (NSS)**				
1/2	1.03 ± 0.50	1.37 ± 0.40	0.23 ± 0.01 a	10.30 ± 0.90 a
1/4	1.03 ± 0.50	1.33 ± 0.14	0.22 ± 0.03 a	9.30 ± 1.00 b
1/8	1.03 ± 0.50	1.13 ± 0.15	0.19 ± 0.04 b	7.70 ± 0.80 c
Significance				
G	***	NS	**	*
NSS	NS	NS	**	***
G * NSS	NS	NS	**	NS

Significance: ***, **, and * respectively for *p* ≤ 0.001, *p* ≤ 0.01, and *p* ≤ 0.05; NS, not significant. Means values (± standard deviation) within each column and main effect followed by different letters are significantly different, according to SNK test (*p* = 0.05).

**Table 4:** Main effects of genotypes and nutrient solution strength on development stage, substrate coverage, substrate uniformity and density of microgreens (first experiment).

	Development Stage ^(1)^	Substrate Coverage ^(2)^	Substrate Uniformity ^(3)^	Density
	1-3	1-5	1-3	Microgreens cm^−2^
**Genotype** (G)				
Broccoli raab	3.0 ± 0.1	2.2 ± 0.4 a	2.0 ± 0.0 a	4.1 ± 0.5
Broccoli	3.0 ± 0.1	2.0 ± 0.1 a	1.8 ± 0.4 ab	4.3 ± 1.5
Cauliflower	3.0 ± 0.1	1.6 ± 0.5 b	1.4 ± 0.5 b	3.7 ± 0.5
**Nutrient solution strength (NSS)**				
1/2	3.0 ± 0.1	2.1 ± 0.6	1.8 ± 0.4	3.9 ± 0.5
1/4	3.0 ± 0.2	1.9 ± 0.3	1.7 ± 0.5	4.0 ± 1.4
1/8	3.0 ± 0.1	1.8 ± 0.4	1.8 ± 0.4	4.1 ± 0.6
Significance				
G	NS	**	*	NS
NSS	NS	NS	NS	NS
G * NSS	NS	NS	NS	NS

^(1)^ Development stage: 1—cotyledonary leaves; 2—true leaves (≤5 mm); 3—true leaves (>5mm). ^(2)^ Substrate coverage: 1—low; 2—good; 3—excessive. ^(3)^ Substrate uniformity: 1—not uniform in the centre; 2—uniform; 3—not uniform along the side. Significance: **, and * respectively for *p* ≤ 0.01, and *p* ≤ 0.05; NS, not significant. Means values (± standard deviation) within each column and main effect followed by different letters are significantly different, according to SNK test (*p* = 0.05).

**Table 5:** Main effects of genotypes and the NH_4_:NO_3_ ratio on the number and length of true leaves, yield and height of microgreens (second experiment).

	True Leaves	Leaves Length	Yield	Seedling Height
	Number Seedling^−1^	cm	g cm^−2^	cm
**Genotype** (G)				
Broccoli raab	2.00 ± 0.10 a	1.39 ± 0.10 a	0.21 ± 0.02 b	9.09 ± 0.27 b
Broccoli	1.00 ± 0.10 b	0.40 ± 0.01 b	0.22 ± 0.03 b	9.84 ± 0.19 a
Cauliflower	1.00 ± 0.10 b	0.40 ± 0.01 b	0.29 ± 0.03 a	9.78 ± 0.48 a
**NH_4_:NO_3_(%)** (R)				
5–95	1.33 ± 0.05	0.71 ± 0.46	0.23 ± 0.04	9.46 ± 0.65
15–85	1.33 ± 0.05	0.75 ± 0.53	0.25 ± 0.05	9.63 ± 0.35
25–75	1.33 ± 0.05	0.73 ± 0.50	0.24 ± 0.05	9.62 ± 0.47
Significance				
G	***	***	***	*
R	NS	NS	NS	NS
G * R	NS	NS	NS	NS

Significance: ***, and * respectively for *p* ≤ 0.001 and *p* ≤ 0.05; NS, not significant. Means values (± standard deviation) within each column and main effect followed by different letters are significantly different, according to SNK test (*p* = 0.05).

**Table 6:** Main effects of genotypes and NH_4_:NO_3_ ratio on dry matter and inorganic anion of microgreens (second experiment).

	Dry Matter	Cl^−^	NO_3_^−^	SO_4_^2−^	Na^+^	Mg^2+^	K^+^	Ca^2+^
	g 100 g^−1^ FW		g 100 g^−1^ DW					
**Genotype (G)**								
Broccoli raab	6.29 ± 0.23	0.97 ± 0.25	8.03 ± 1.34 b	2.18 ± 0.18 c	0.16 ± 0.03 b	0.30 ± 0.03	2.23 ± 0.31 b	1.08 ± 0.11
Broccoli	5.81 ± 1.01	0.89 ± 0.31	8.29 ± 1.25 b	2.45 ± 0.36 b	0.15 ± 0.02 b	0.30 ± 0.02	2.51 ± 0.17 a	1.10 ± 0.11
Cauliflower	6.53 ± 1.41	0.91 ± 0.33	9.57 ± 0.55 a	2.80 ± 0.26 a	0.18 ± 0.03 a	0.30 ± 0.02	2.27 ± 0.29 b	1.06 ± 0.11
**NH_4_:NO_3_** (%) **(R)**								
5–95	6.45 ± 0.32	0.78 ± 0.15 b	8.22 ± 1.50 b	2.36 ± 0.35 b	0.18 ± 0.02	0.32 ± 0.01	2.12 ± 0.29 c	1.16 ± 0.08 a
15–85	6.04 ± 0.94	0.74 ± 0.14 b	9.55 ± 0.59 a	2.68 ± 0.38 a	0.14 ± 0.01	0.30 ± 0.02	2.37 ± 0.21 b	1.08 ± 0.09 ab
25–75	6.09 ± 1.47	1.25 ± 0.24 a	8.13 ± 1.08 b	2.38 ± 0.34 b	0.17 ± 0.04	0.29 ± 0.03	2.52 ± 0.18 a	1.01 ± 0.11 b
Significance								
G	NS	NS	***	***	*	NS	*	NS
R	NS	**	**	**	NS	NS	***	*
G*R	**	NS	**	NS	**	NS	NS	NS

Significance: ***, **, and * respectively for *p* ≤ 0.001, *p* ≤ 0.01, and *p* ≤ 0.05; NS, not significant. Means values (± standard deviation) within each column and main effect followed by different letters are significantly different, according to SNK test (*p* = 0.05).

**Table 7:** Effects of genotypes and NH_4_:NO_3_ ratio on fiber, protein, α-tocopherol and β-carotene content (second experiment).

	Fiber	Protein	α-Tocopherol	β-Carotene
	g 100 g^−1^ FW		mg 100 g^−1^ FW	
**Genotype (G)**				
Broccoli raab	0.355 ± 0.220	2.35 ± 0.21 b	2.02 ± 0.59 b	3.57 ± 0.95 b
Broccoli	0.517 ± 0.095	2.34 ± 1.09 b	5.08 ± 2.47 b	5.35 ± 1.54 a
Cauliflower	0.681 ± 0.259	3.12 ± 0.32 a	10.45 ± 7.71 a	6.48 ± 2.43 a
**NH_4_:NO_3_** (%) **(R)**				
5–95	0.493 ± 0.073	2.31 ± 0.19	4.29 ± 3.76	4.37 ± 1.09 b
15–85	0.459 ± 0.229	2.40 ± 0.21	4.98 ± 4.64	4.60 ± 1.14 b
25–75	0.600 ± 0.337	3.01 ± 1.01	7.86 ± 7.58	6.29 ± 2.69 a
Significance				
*G*	NS	**	*	**
R	NS	NS	NS	**
G*R	NS	*	NS	NS

Significance: **, and * respectively for *p* ≤ 0.01, and *p* ≤ 0.05; NS, not significant. Means values (± standard deviation) within each column and main effect followed by different letters are significantly different, according to SNK test (*p* = 0.05).
